# Structural healing after rotator cuff repair: clinical predictors, biomarkers, prediction models, and readiness for precision care

**DOI:** 10.3389/fmed.2026.1955208

**Published:** 2026-09-14

**Authors:** Qin Zhou, Xu Chen, Xiaoling Luo, Lingling Zhang, Hongyan Zhu, Yanping He, Mei Yao, Peifang Li

**Affiliations:** Department of Orthopedic Surgery, West China Hospital, Sichuan University/West China School of Nursing, Sichuan University, Chengdu, China

**Keywords:** biomarker, clinical predictor, precision medicine, prediction model, quantitative imaging, risk stratification, rotator cuff repair, structural healing

## Abstract

Structural healing after rotator cuff repair is clinically important, yet individual healing trajectories remain difficult to predict, monitor, and translate into individualized care. Evidence spans routine clinical predictors, systemic metabolic biomarkers, quantitative imaging, tissue phenotypes, genetic variation, transcriptomic signatures, and artificial intelligence–based prediction models. However, prior reviews rarely distinguish these evidence objects or evaluate them according to intended clinical use and stage of validation. This focused Mini Review assesses readiness for precision care across five linked dimensions: measurement validity, context-specific clinical validity, added value beyond routine predictors, independent validation, and action or impact evidence. Current literature is dominated by prognostic associations and model-development studies, whereas routine clinical predictors remain the most mature benchmarks. Promising signals have emerged across modalities, but heterogeneous structural endpoints, inconsistent measurement procedures, limited external validation, and the absence of decision-linked impact studies continue to impede individualized care. The principal challenge is therefore not candidate scarcity, but conversion of associations into reproducible, transportable, and actionable evidence. By mapping each evidence class to prognosis, monitoring, diagnosis, or treatment-effect prediction, this review proposes a translational roadmap for biomarker-informed risk stratification, postoperative surveillance, and prospective evaluation of individualized management pathways.

## 1 Introduction

Rotator cuff repair aims to restore continuity between the torn tendon and its humeral insertion. After fixation, structural healing depends on inflammation, matrix deposition, vascular adaptation, and remodeling at the tendon-to-bone interface. Failure of this process may appear as incomplete healing or retear on ultrasound or magnetic resonance imaging. Repair integrity is associated with better pain and function, although imaging does not fully explain clinical recovery (1). Patients may improve across structural categories, while better integrity accompanies favorable muscle-atrophy and fatty-infiltration trajectories (2). Structural healing is therefore clinically meaningful, but it is not a complete surrogate for recovery.

Research on structural healing initially centered on clinical and anatomical determinants. Age, tear size, retraction, fatty infiltration, bone mineral density, and work activity underpin the Rotator Cuff Healing Index (RoHI) (3, 4). In parallel, biochemical, genetic, histologic, and imaging characteristics have been investigated as indicators of tendon condition and repair biology (5). In this setting, a biomarker is a defined characteristic measured as an indicator of a biological process or response (6). Biomarkers of structural healing may therefore reflect systemic metabolism, local tendon and muscle quality, molecular responses, or postoperative tissue changes.

The field has now progressed from isolated candidate discovery toward multimodal and longitudinal assessment. Systemic measures are being studied alongside quantitative magnetic resonance imaging, ultrasonography, elastography, histology, genetic variation, and transcriptomic signatures. Radiomics and machine learning can also combine clinical and image-derived data into individualized risk estimates. However, these approaches occupy different points in the research pathway. Much of the evidence remains associative or limited to model development, with variation in sampling time, imaging protocol, structural endpoint, follow-up window, and validation strategy. Current research is therefore moving from signal identification toward reproducible and clinically interpretable measurement.

This shift changes the central question from whether a candidate is associated with healing to how and when it could be used. Preoperative prognosis, postoperative monitoring, diagnosis of structural failure, and treatment-effect prediction each require different study designs and performance criteria. Likewise, high discrimination from a prediction algorithm does not establish biomarker validity or clinical benefit (7–9). This focused Mini Review examines human evidence for clinical predictors, biomarkers, and prediction models of structural healing or retear after rotator cuff repair. It evaluates how each evidence object could contribute to precision care, the stage reached within its intended context of use, and the validation required before it can support individualized clinical decisions. The object–use framework applied throughout this review is summarized in Figure 1.

**Figure 1:**
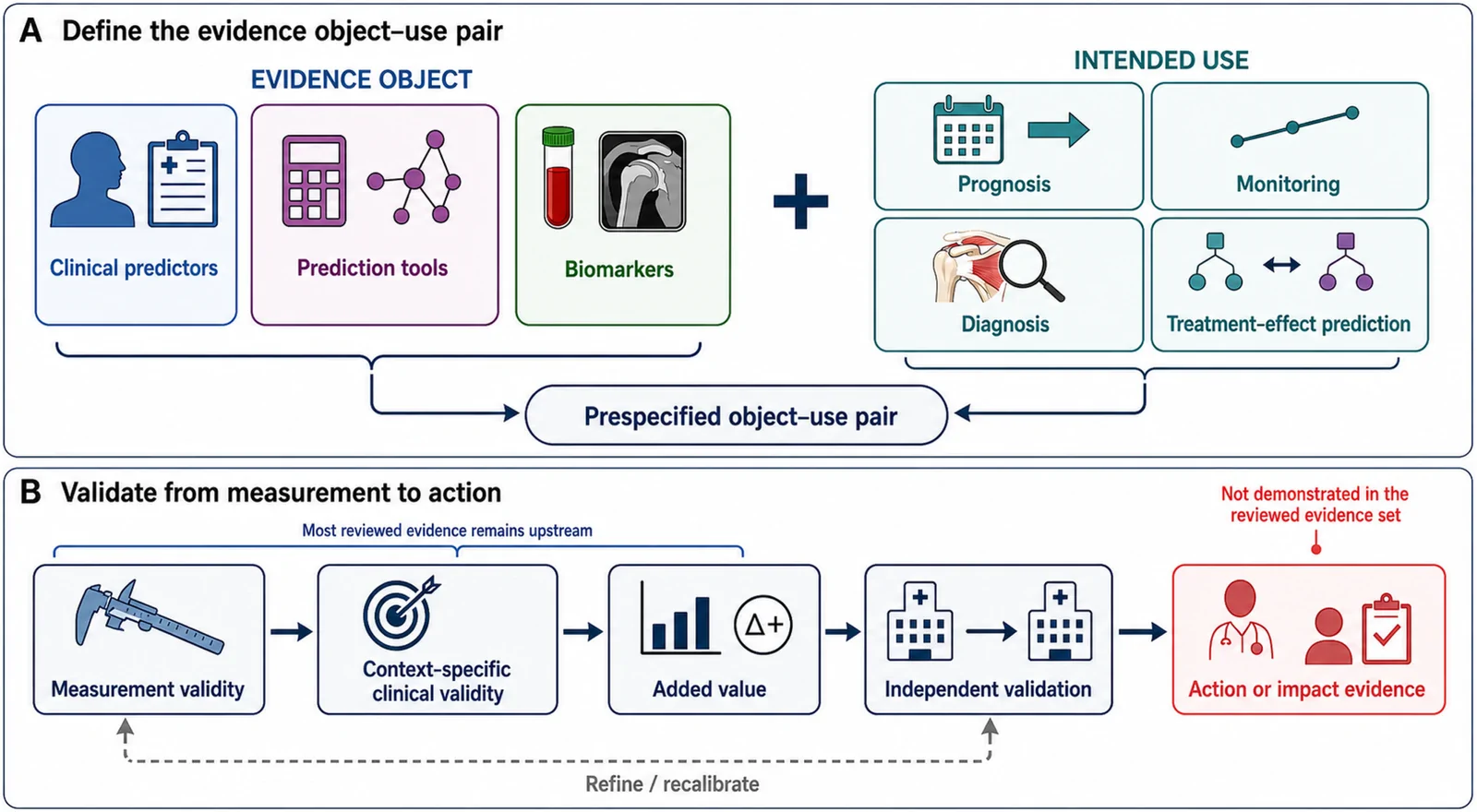
Object–use framework for evaluating readiness for precision care after rotator cuff repair. **(A)** A clinical predictor, prediction tool, or biomarker is paired with a prespecified use: prognosis, monitoring, diagnosis, or treatment-effect prediction. **(B)** Each object–use pair is evaluated across five dimensions: measurement validity, context-specific clinical validity, added value beyond routine predictors, independent validation, and action or impact evidence. The bracketed annotation and red terminal box summarize the maturity of the reviewed evidence, while the dashed arrow denotes refinement or recalibration after external evaluation.

## 2 What makes evidence ready for precision care?

A biomarker is a defined characteristic measured as an indicator of a biological process or response. Its meaning depends on a stated context of use (6). Clinical predictors may include nonbiological attributes such as age or work activity. Prediction scores and machine-learning algorithms combine inputs to estimate an outcome; they are tools rather than biomarkers. Quantitative imaging features may function as imaging biomarkers when they represent reproducible biological characteristics. Glycated hemoglobin (HbA1c) is best treated as a systemic metabolic biomarker and modifiable exposure marker, not as a direct measure of local tendon repair.

This review evaluates readiness for precision care across five linked dimensions: measurement validity, context-specific clinical validity, added value beyond routine clinical and imaging predictors, independent validation, and action or impact evidence. The distinctions among evidence objects, intended uses, and validation dimensions are summarized in Figure 1.

Context-specific clinical validity replaces a single structural-association requirement. For prognosis, the candidate must estimate a future structural outcome at a clinically relevant time. For monitoring, a reproducible within-person change must precede and inform a subsequent outcome. For diagnosis, performance requires sensitivity, specificity, and comparison with an appropriate reference standard. Treatment-effect prediction requires evidence that the effect of an intervention differs across marker-defined groups. A prognostic association alone cannot establish treatment selection.

Measurement validity also differs by evidence object. Clinical scores require explicit variable definitions, reproducible grading, and reliable handling of missing inputs. Imaging biomarkers require test–retest repeatability, inter-reader and segmentation agreement, and stability across scanners and acquisition protocols. Laboratory markers require defined collection windows, assay precision, within-person biological variation, and reproducible thresholds. Tissue and omics studies require standardized sampling location, preanalytic handling, technical replication, and control of batch effects. These object-specific requirements and the class-level readiness profiles are summarized in Table 1, whereas the study-level evidence atlas is provided in Supplementary Table S1.

**Table 1:** Context-specific readiness for precision care across evidence classes relevant to structural healing after rotator cuff repair.

Evidence class and intended use	Measurement validity	Context-specific clinical validity	Added value	Independent validation	Action or impact evidence	Key gap and priority next step	Key references
Clinical predictors and multivariable scores; mainly prognosis	Partial	Established	Partial	Partial	Not demonstrated	Key gap: Repeated predictors and one geographic RoHI evaluation; broad calibration and action evidence remain absent. Priority next step: Fixed-score multicenter recalibration → threshold net benefit → strategy trial.	(3, 4, 10, 11, 30)
AI and radiomics models; prognosis	Unclear	Partial	Not demonstrated	Partial	Not demonstrated	Key gap: Strong internal performance and one external AI test; no locked routine-model comparison. Priority next step: Locked patient-level pipeline → routine comparator → independent calibration → decision curves.	(7–9, 12–14)
Quantitative MRI and CEUS perfusion biomarkers and preoperative MRI signs; prognosis	Partial	Partial	Not demonstrated	Not demonstrated	Not demonstrated	Key gap: Protocols, thresholds, and candidate definitions remain unharmonized and unvalidated across MRI and CEUS. Priority next step: Harmonized acquisition → repeatability → prespecified threshold → independent prognostic study.	(15–17, 29, 34)
Quantitative MRI, ultrasound, and elastography biomarkers; monitoring	Partial	Partial	Not demonstrated	Not demonstrated	Not demonstrated	Key gap: No repeatable within-person change threshold has been linked to a later structural outcome. Priority next step: Multicenter repeatability → prespecified landmark/change threshold → later-outcome validation.	(27–29)
Postoperative MRI sign; diagnosis	Partial	Unclear	Not demonstrated	Not demonstrated	Not demonstrated	Key gap: No independent accuracy study has separated the index sign from the target condition. Priority next step: Prespecified sign → blinded diagnostic-accuracy study → independent validation.	(34)
Systemic metabolic biomarkers and exposure markers; prognosis, including postoperative landmark prognosis	Partial	Unclear	Not demonstrated	Not demonstrated	Not demonstrated	Key gap: Two HbA1c cohorts reported adverse prognostic associations, but landmark windows differed; lipid and vitamin D findings were inconsistent. Priority next step: Prespecified sampling and landmark → routine-predictor comparison → external prognostic validation.	(21–26, 31–33)
Tissue and histologic biomarkers; prognosis	Unclear	Unclear	Not demonstrated	Not demonstrated	Not demonstrated	Key gap: Sampling and scoring vary; positive and null studies do not replicate one fixed candidate. Priority next step: Standardized sampling + blinded reliability → candidate-specific replication → clinical benchmark.	(37–40)
Genetic and transcriptomic biomarkers; prognosis	Unclear	Partial	Not demonstrated	Not demonstrated	Not demonstrated	Key gap: Discovery associations exist; no locked signature, same-variant replication, or clinical benchmark. Priority next step: Lock signature → ancestry-aware and batch-robust independent test → routine benchmark.	(35, 36, 41–44)

Readiness-rating definitions: Established = consistent direct evidence for the same use and dimension, generally from at least two independent cohorts; Partial = direct evidence supports at least one necessary component, but replication or another essential requirement is missing; Unclear = materially conflicting findings or insufficient reporting; Not demonstrated = no included study directly supports any necessary component of the dimension.

Use-specific aggregation: Table 1 reports class–use judgments. Ratings were derived from all information reported for the relevant profiles in Supplementary Table S1. Evidence was not pooled across uses or calculated by averaging the profile-level status entries.

1. Table 1 reports class-level judgments. A favorable result from one study does not establish readiness for an entire evidence class.

2. “Independent validation” requires a prespecified and unchanged candidate, measurement process, model, or threshold evaluated in a new population or setting. Split-sample evaluation or within-cohort modification is not independent validation.

3. “Added value” requires comparison with a prespecified benchmark containing routine clinical and conventional imaging predictors in the same evaluation cohort.

4. “Treatment-effect prediction” requires a prespecified candidate-by-treatment interaction. No qualifying evidence was identified among the included studies.

5. Biomarker and context-of-use terminology follows BEST (6). Model and radiomics evaluation is informed by TRIPOD + AI, PROBAST + AI, and current radiomics guidance (7–9).

6. AI, artificial intelligence; MRI, magnetic resonance imaging.

High discrimination does not complete the framework. Prediction-model reporting requires transparent populations, predictors, outcomes, partitioning, missing-data handling, calibration, and external evaluation (7). Model appraisal must distinguish development quality from risk of bias in performance evaluation (8). Radiomics adds requirements for acquisition, segmentation, feature definition, comparator testing, and clinical evaluation (9). Added value should be tested against a locked routine-variable model in the same population. Independent validation should then examine calibration, discrimination, threshold performance, and clinical net benefit in a new setting.

PubMed/MEDLINE and Europe PMC were searched on 29 July 2026, with no lower publication-date limit. Search concepts combined rotator cuff repair and structural healing or retear with terms related to clinical predictors and prediction models, molecular and omics biomarkers, quantitative magnetic resonance imaging, ultrasonography, elastography, radiomics, perfusion, and tissue characteristics. Peer-reviewed human studies of adults undergoing rotator cuff repair were eligible when a clinical predictor, prediction model, molecular marker, or imaging-derived measure was evaluated in relation to postoperative structural healing, nonhealing, or retear. Supplementary Table S1 presents a representative selection across evidence classes and intended clinical uses, prioritizing methodologically informative studies while retaining important positive, negative, and discordant findings.

Readiness was assessed separately for each evidence class within a defined clinical use: prognosis, monitoring, diagnosis, or treatment-effect prediction. Table 1 therefore presents class–use judgments, and evidence was not pooled across uses. Measurement standardization and reliability informed measurement validity. Context-specific clinical validity required direct evidence appropriate to the stated use: association with a future structural outcome for prognosis, repeatable within-person change preceding a later structural outcome for monitoring, accuracy against a structural reference standard for diagnosis, or a prespecified candidate-by-treatment interaction within a comparative design for treatment-effect prediction. Direct comparison with routine predictors informed added value. Evaluation of a prespecified candidate in a new cohort or setting informed independent validation. Decision-linked prospective evaluation informed action or impact evidence. Established generally required consistent direct evidence from at least two independent cohorts for the same use and dimension. Partial indicated that direct evidence supported at least one necessary component, but replication or another essential requirement was missing. Unclear indicated materially conflicting findings or insufficient reporting. Not demonstrated indicated that no included study directly supported any necessary component of the dimension. The class-level ratings were derived from all information reported for the relevant profiles in Supplementary Table S1 rather than by averaging the profile-level status entries.

## 3 Current evidence across predictors, biomarkers, and models

Representative study-level evidence, including positive, negative, and conflicting findings, is detailed in Supplementary Table S1.

### 3.1 Clinical predictors and prediction tools

Routine variables are the benchmark for any additional prognostic test. Age and tear size showed robust associations in a multicenter cohort (3). RoHI combines these predictors with retraction, fatty infiltration, bone mineral density, and work activity. Failure increased from 6.0% at scores of four or lower to 55.2% at five or higher and 86.2% at ten or higher (4). RoHI should therefore be described as a multivariable prognostic score. Most inputs are routine, although bone mineral density may affect implementation.

A geographic evaluation in 133 Thai patients produced an area under the curve (AUC) of 0.780. A locally modified score reached 0.827, although the difference was not significant (10). Because the modification was developed and assessed in the same cohort, the original RoHI result provides the cleaner transport estimate. A subscapularis-specific index illustrates the appeal of simple scores, but its current evidence comes from a development cohort with incomplete imaging follow-up (11). The next evaluation of these scores should retain prespecified variables and thresholds, report calibration, and compare clinical net benefit across plausible decisions.

Machine-learning studies evaluate prediction algorithms rather than a distinct biomarker class. A graph model trained on 1,631 repairs achieved 96.93% internal accuracy and 79.55% accuracy in an external cohort of 518 cases (12). The difference between development and external performance may reflect prevalence, case mix, thresholds, or measurement practice. Clinical interpretation of the external result requires calibration and threshold-specific measures in addition to accuracy.

MRI radiomics and arthroscopic-image models provide related evidence. In one radiomics study, retear prediction reached an AUC of 0.923 in development and 0.790 across repeated 20-patient internal validation subsets; a combined model was approximately 0.81 (13). A DenseNet model using 1,394 intraoperative images from 580 patients reported a test AUC of 0.92 within the source dataset (14). These findings support image-derived predictive signal. The clinically relevant comparison is the added performance of a locked image-based model over age, tear morphology, muscle quality, diabetes, and standard MRI interpretation.

Event scarcity and image-level partitioning can increase overfitting or leakage risk. Patient-level separation, locked preprocessing, and transparent handling of multiple images per patient are therefore essential (7, 8). Measurement validity also requires segmentation agreement and feature stability across acquisition settings. Comparisons should use the same external cohort and routine-variable baseline because scanner, surgeon, and site signatures can contribute to apparent performance (9).

### 3.2 Quantitative imaging biomarkers and image-derived predictors

Quantitative MRI can create continuous tendon and muscle phenotypes. Two-point Dixon MRI reported muscle-specific fat-fraction AUCs from 0.82 to 0.94 in a retrospective matched sample (15). T2 mapping differentiated healing status in large or massive tears when conventional Goutallier grades did not (16). A study of medium-sized tears identified a tendon-stump C/D signal ratio associated with healing failure, with an internally derived AUC of 0.82 (17). The ratio is an imaging biomarker candidate because it quantifies a tissue characteristic. Its measurement evidence should include reader agreement, segmentation rules, scanner stability, and threshold reproducibility before transport evaluation.

Static bony morphology is better classified as an image-derived clinical predictor. A small cohort associated higher critical shoulder angle and glenoid inclination with full-thickness retear (18). Later cohorts found no association between critical shoulder angle and retear (19), including a series of 397 repairs (20). The differing estimates make standardized measurement and multicenter evaluation more informative than treating shoulder geometry as a biomarker class.

### 3.3 Systemic metabolic biomarkers and exposure markers

Baseline metabolic measures reflect systemic exposures rather than local tendon biology. Low high-density lipoprotein was associated with retear in a large or massive tear subgroup (21). Preoperative lipid control was associated with lower retear, although the control category may also capture adherence and other health behaviors (22). A 620-patient cohort found no association between serum lipids or statin use and retear (23). Another study linked missed statin therapy and an atherosclerotic risk score to failure, which again highlights treatment-confounding and healthy-adherer effects (24).

Vitamin D findings also vary. One retrospective cohort reported retear in 26.67% of deficient patients and 9.09% of nondeficient patients (25). An earlier prospective cohort, in which 88% of participants were deficient, found no association with postoperative integrity (26). Exposure range, assay timing, thresholds, and confounding can influence these estimates. Future evaluation should prespecify the biological exposure window and compare added prognostic value with routine clinical variables before testing a management strategy.

### 3.4 Longitudinal monitoring, postoperative landmark prognosis, and diagnosis

Quantitative imaging can characterize a changing repair rather than only baseline risk. Ultrashort echo-time T2* mapping described a tendon trajectory that approached healthy-control values by 12 months (27). Shear-wave elastography showed spatial and temporal increases in repaired-tendon stiffness (28). Contrast-enhanced ultrasound in 20 patients linked lower preoperative supraspinatus perfusion with retear and showed improved perfusion after repair (29). These studies document longitudinal biological change but do not establish monitoring validity. Such validity requires a repeatable within-person change measured at a prespecified landmark and associated with a subsequent structural outcome.

In 1,526 repairs, a multivariable model including tear area, age, tear type, hospital type, and six-week forward flexion and external rotation predicted six-month ultrasound integrity (AUC 0.84), versus 0.77 for tear area alone (30). This supports time-updated prediction, but the incremental contribution of the six-week motion measures beyond the remaining predictors was not isolated; independent external validation and calibration are required.

Postoperative HbA1c is a clinically accessible systemic metabolic biomarker and modifiable exposure marker. Among 103 patients with diabetes, retear occurred in 51.1% of those whose HbA1c increased during postoperative months three to six, compared with 14.3% without an increase. The adjusted odds ratio was 5.402 (31). Another cohort found retear in 50.0% of patients with uncontrolled six-month HbA1c, versus 8.7% with controlled diabetes and 16.8% without diabetes (32). A smaller study found no significant structural difference between HbA1c-defined groups (33).

Together, these cohorts support time-updated prognostic associations, but they do not establish monitoring validity. HbA1c measurement should occur at a prespecified landmark before the structural assessment. Repairs that have already failed should be separated from those still at risk. The analysis should define the HbA1c trajectory, address informative follow-up and reverse causation, and distinguish prognostic information from the effect of changing glycemic exposure.

Subcoracoid cyst findings were associated with retear, but the study mixed preoperative prognostic and postoperative diagnostic signals (34). A separate blinded diagnostic-accuracy study is needed.

### 3.5 Tissue, genetic, and transcriptomic biomarkers

Tissue and molecular studies connect structural healing with local biology. Candidate-gene studies linked tenascin-C haplotypes and an ESRRB variant with healing failure (35, 36). These discovery analyses define candidates for prespecified replication. Clinical evaluation should also test whether a genetic signal adds information beyond tear morphology and remains stable across ancestry groups.

Histologic findings differ across cohorts. Severe supraspinatus degeneration was associated with retear in one 187-patient cohort (37). A 56-patient study found no difference in degeneration score between healed and failed repairs (38). A prospective cohort found that gross tendon appearance and Bonar histopathology did not predict outcome (39). A prospective muscle-biopsy cohort also found no significant association between measured fatty accumulation, atrophy, or fibrosis and retear, with seven retears observed (40). A common sampling site, blinded scoring, inter-reader agreement, and quantitative assay reproducibility would make these results more directly comparable.

Transcriptomic studies reveal pathway-level heterogeneity. A small case-control analysis found pathway differences without a significant individual gene; its composite poor-outcome definition included structural failure (41). RNA sequencing separated clusters with different healing rates, although the clusters also differed in tear size and retraction (42). A multicenter discovery cohort of 25 patients associated intact healing with extracellular-matrix stabilization and lower inflammatory signatures (43). MMP-3 and TIMP-1 expression were higher among six retears in an earlier study (44). These studies support biological hypotheses that can be converted into locked signatures and tested in independent cohorts.

Samples taken during surgery may characterize chronic disease, mechanical damage, or local inflammation as well as healing capacity. A molecular signature should therefore be evaluated after accounting for tear size, retraction, and muscle degeneration. Translation also requires a feasible preoperative specimen or a defined intraoperative decision, together with batch-robust processing and a prespecified analysis pipeline.

### 3.6 Treatment-effect prediction requires interaction evidence

The studies above principally estimate prognosis, describe healing biology, or develop monitoring hypotheses. Treatment-effect prediction asks a different question: whether the effect of a surgical, rehabilitation, imaging, or metabolic strategy differs across marker-defined groups. The appropriate analysis is a prespecified marker-by-treatment interaction within a comparative design. Once a reproducible interaction is shown, an impact study can test whether acting on that information improves structural and patient-reported outcomes.

## 4 Discussion: from association to precision care

As summarized in Table 1, the available literature supports distinct translational profiles rather than a single hierarchy of candidate maturity. Clinical predictors and multivariable scores currently provide the most developed basis for individualized risk estimation, whereas quantitative imaging, systemic metabolic measures, and tissue or molecular assays offer complementary biological information but remain less validated. Prediction algorithms should be evaluated as analytical tools rather than treated as biomarkers. This distinction is central to precision care because individualized management requires not only accurate risk estimates, but also reproducible measurement, transportable performance, and evidence that the information can support a defined clinical decision.

RoHI and postoperative HbA1c illustrate two different routes toward precision care. RoHI is a pragmatic score built mainly from routine preoperative inputs (4, 10). Its next step is fixed-score multicenter validation, recalibration of absolute risk, and evaluation of thresholds linked to concrete decisions such as surveillance intensity or enrollment in risk-stratified trials. HbA1c is a systemic metabolic biomarker and modifiable exposure marker with time-updated prognostic associations (31–33). Future studies should prespecify sampling landmarks, distinguish prognostic information from the effect of glycemic modification, and test whether a biomarker-informed metabolic-management pathway improves structural healing, safety, adherence, and patient-reported outcomes. Neither marker currently supports routine treatment selection.

Measurement validity differs by class. Imaging requires test–retest repeatability, reader and segmentation agreement, and scanner stability; tissue studies need standardized sampling and blinded scoring; omics need batch control and locked signatures. Laboratory monitoring additionally requires defined sampling windows, within-person variation, and reproducible thresholds.

Advanced imaging and AI models should lock acquisition and preprocessing, prespecify structural outcomes and follow-up, keep all images from one patient in the same partition, and benchmark against routine predictors. Reports should include calibration, discrimination, threshold performance, and net benefit. Clinical value requires improvement beyond that baseline.

Independent validation should challenge the development setting. Participating centers should vary in scanner, surgeon, rehabilitation pathway, and case mix. A model can then be recalibrated transparently if ranking remains useful but absolute risks shift. Prespecified subgroup analyses can examine diabetes, sex, tear size, and access to follow-up without training separate unstable models. Decision curves should use thresholds that correspond to realistic clinical actions.

Precision care also requires alignment between the predicted structural outcome and outcomes that matter to patients. Repair integrity is clinically meaningful, but it is not a complete surrogate for pain, function, return to activity, revision risk, or treatment burden. Prospective impact studies should therefore test whether marker-informed decisions—such as intensified imaging surveillance, targeted metabolic optimization, modified rehabilitation, or enrollment in risk-stratified comparative studies—improve both structural and patient-reported outcomes without unnecessary intervention. These pathways are priorities for prospective testing rather than currently validated recommendations.

## 5 Conclusion

Clinical predictors, prediction tools, and biomarkers provide complementary but non-interchangeable information about structural healing after rotator cuff repair. The five-dimension framework links each evidence object to a defined context of use and clarifies the requirements for precision care: reproducible measurement, context-specific clinical validity, added value beyond routine predictors, independent validation, and evaluation within a prespecified clinical action. Current evidence supports individualized risk-stratification research more strongly than routine biomarker-guided management. The next phase should therefore prioritize multicenter validation and prospective impact studies that test whether marker-informed surveillance, metabolic optimization, rehabilitation, or trial enrollment improves both structural and patient-reported outcomes.
