# Supplementary material for: Structural healing after rotator cuff repair: clinical predictors, biomarkers, prediction models, and readiness for precision care
Source: Front Med (Lausanne). 2026 Sep 14;13:1955208. doi: 10.3389/fmed.2026.1955208 (PMC13617384; doi:10.3389/fmed.2026.1955208)
Supplement: Supplementary file 1 [file Table1.docx]

Supplementary Table S1. Representative evidence profiles for clinical predictors, biological biomarkers, and prediction models of structural healing after rotator cuff repair.

| **Evidence object / candidate** | **Intended use** | **Evidence base; timing → structural endpoint*** | **Main signal*** | **Critical boundary** | **Evidence-profile status** | **Priority for further evaluation** | **Key references** |
| --- | --- | --- | --- | --- | --- | --- | --- |
| Clinical predictor / age + tear size | Prognosis | Multicenter cohort; n=217 imaged; pre-/intraoperative → 12-month imaging | Healing 66–68% in small/medium tears; 47% in large; 27% in massive tears. Age was adverse. | Benchmark, not a biomarker. No decision threshold. | **Established** | Lock a multicenter baseline model. Report calibration and net benefit. | Rashid 2017 (3); Kwon 2019 (4) |
| Clinical score / Rotator Cuff Healing Index (RoHI) | Prognosis | Derivation n=603; imaging at ≥12 months. Geographic evaluation n=133; imaging at 6–24 months. | Strong risk gradient. Original RoHI AUC 0.780 in Thailand. | Single-center derivation. BMD may limit use. Local modification was not independently validated. | **Partial** | Validate the fixed score across centers. Recalibrate absolute risk. Test decision thresholds. | Kwon 2019 (4); Manop 2023 (10) |
| Clinical score / Subscapularis Healing Index | Prognosis | 279 imaged among 931 eligible; preoperative → ≥2-year ultrasound | Failure was 4% at ≤4 points and 55% at ≥9 points. | Subscapularis-specific. Selective imaging follow-up. Development only. | **Partial** | Test the locked score in an independent cohort with complete imaging follow-up. | Kilic 2024 (11) |
| Dynamic clinical model / routine factors + tear area + 6-week range of motion | Landmark prognosis | Cohort n=1,526; intraoperative + 6 weeks → 6-month ultrasound | Full multivariable model AUC 0.84 versus 0.77 for tear area alone. | Internal result. Short endpoint. Single repair construct. | **Partial** | Independently validate the landmark model. Add later imaging and calibration. | Guo 2022 (30) |
| AI prediction model / EV-GCN using 62 variables | Prognosis | Derivation n=1,631; external n=518; preoperative → postoperative retear | Accuracy fell from 96.93% internally to 79.55% externally. | Accuracy dominated reporting. Calibration and threshold utility were absent. High input burden. | **Partial** | Run a locked independent test. Report calibration, threshold metrics, and decision curves. | Zhang 2024 (12) |
| Radiomics model / MRI signature ± clinical factors | Prognosis | n=101; 26 retears; repeated 20-patient internal validation subsets; preoperative MRI → 6–12-month MRI | AUC 0.923 in development and 0.790 in internal validation; combined model approximately 0.81. | Small internal validation subsets. A data-selected clinical-only comparator was included; no prespecified locked baseline or independent validation. | **Partial** | Lock segmentation and features. Test against a routine model in a multicenter cohort. | Fei 2024 (13) |
| Image-based AI model / intraoperative arthroscopic DenseNet | Prognosis | 580 patients; 1,394 images; immediate post-repair image → MRI within 2 years | Internal test AUC 0.92. | Intraoperative use only. The reported partitioning information was insufficient to exclude image-level leakage. No independent comparison was performed. | **Partial** | Use patient-level independent validation. Compare with a locked clinical/intraoperative baseline. | Cho 2023 (14) |
| Quantitative MRI biomarker / tendon-stump C/D signal ratio | Prognosis | Single-center cohort n=136; preoperative MRI → postoperative Sugaya status | Cutoff 1.09; AUC 0.82; adjusted OR 11.52. | Medium tears only. The measure was sequence dependent, and evidence for inter-reader reliability and scanner stability was insufficient. | **Partial** | Standardize ROI placement. Test reader/scanner stability. Independently test the cutoff. | Kim 2026 (17) |
| Quantitative MRI biomarker / two-point Dixon muscle fat fraction | Prognosis | 91 repaired shoulders: 41 failed, 50 intact; plus 45 conservative controls; preoperative MRI → postoperative MRI | Muscle-specific AUCs ranged from 0.82 to 0.94. | Selection spectrum. Protocol-specific cutoffs. No independent test. | **Partial** | Harmonize acquisition. Test repeatability. Validate locked cutoffs. | Feuerriegel 2024 (15) |
| Quantitative MRI biomarker / supraspinatus and infraspinatus T2 mapping | Prognosis | Prospective cohort n=107; 32 retears; preoperative MRI → 1-year MRI | Higher T2 in retears; Goutallier grades were not significantly different. | Large/massive tears only. Scanner dependent. Thresholds unvalidated. | **Partial** | Use phantom-supported harmonization. Compare with a locked routine baseline. | Iijima 2019 (16) |
| Quantitative MRI biomarker / tendon UTE-T2* trajectory | Monitoring candidate | 25 repairs + 15 controls; 2 retears; 3–24 months → serial MRI/Sugaya | Values rose, then approached control levels by 12 months. | Biological trajectory, not a failure-prediction threshold. Few events. | **Partial (descriptive trajectory only)** | Test repeatability. Prespecify a landmark before a later structural outcome. | Xie 2020 (27) |
| Ultrasound biomarker / shear-wave tendon stiffness | Monitoring candidate | 50 enrolled; 2 week-1 retears excluded; 5 further retears by week 24; stiffness tracked over 1–52 weeks. | Footprint stiffness increased 21% by 6 months. | Descriptive trajectory only. No prespecified stiffness-change threshold or landmark-to-later-failure analysis. Single sonographer and repair technique. | **Partial (descriptive trajectory only)** | Establish multicenter repeatability. Prespecify a change threshold. Test it against later structural imaging. | Hackett 2023 (28) |
| Ultrasound biomarker / CEUS supraspinatus perfusion | Prognosis; monitoring candidate | Prospective pilot n=20; preoperative, 6 months, 1 year → serial ultrasound | Lower baseline perfusion was associated with retear. Perfusion increased after repair. | Pilot sample. Prognostic analyses were unadjusted, and no monitoring threshold linked within-person change to a later structural outcome. | **Prognosis: Partial; monitoring: Partial (biological responsiveness only)** | Standardize CEUS and evaluate prognosis and monitoring in separate prespecified analyses. | Mick 2023 (29) |
| MRI sign / subcoracoid cyst presence or volume change | Prognosis; diagnosis candidate | Retrospective cohort n=109; pre- and postoperative MRI → MRI-defined retear | Preoperative ScC was associated with a higher retear rate (34.8% vs 12.5%), and less postoperative cyst-volume reduction accompanied retear. Reported sensitivity was 72%, specificity 78%, OR 3.73, and ICC 0.91. | The study combined preoperative prognostic and postoperative diagnostic analyses. Diagnostic estimates were reported, but the index sign and reference-standard timing were insufficiently separated. No independent accuracy study was performed. | **Prognosis: Partial; diagnosis: Unclear** | Evaluate the preoperative cyst prospectively. Test the postoperative sign in a separate blinded diagnostic-accuracy study. | Akan 2025 (34) |
| Conventional image-derived predictor / critical shoulder angle and glenoid inclination | Prognosis | Cohorts n=76, n=164, and n=397; preoperative radiograph → postoperative retear ascertainment | Early positive association. Later cohorts were null. | Only 201 MRIs were available; retear ascertainment and follow-up varied across 24 years. | **Unclear** | Standardize measurement in a multicenter reassessment or de-emphasize the candidate. | Garcia 2017 (18); Como 2021 (19); Pang 2026 (20) |
| Systemic metabolic biomarker / postoperative HbA1c trajectory or ≥7% threshold | Landmark prognosis | n=103 with diabetes; n=183 with 35 diabetes; n=51 with diabetes; 3–12-month HbA1c → subsequent or overlapping MRI | Two cohorts reported adverse time-updated prognostic associations. One smaller cohort found no structural association. | In some cohorts, biomarker and outcome windows overlapped, leaving temporal ordering and reverse causation unresolved. Monitoring validity was not evaluated. | **Partial** | Prespecify HbA1c measurement before structural imaging, exclude prior failure, and define the prognostic exposure window and threshold before analysis. | Kim 2023 (31); Yeom 2023 (32); Takahashi 2022 (33) |
| Systemic metabolic biomarkers and exposure markers / HDL, lipid control, statin exposure, ASCVD score | Prognosis | Cohorts n=195, n=148, n=620, and n=182; pre-/perioperative → postoperative MRI/US; 6–12 months where reported | Positive subgroup and adherence-linked signals coexisted with a large null cohort. | Analytes differed. Healthy-user and adherence confounding remain possible. ASCVD is a clinical score; statin use is an exposure. | **Unclear** | Standardize the lipid panel. Record indication and adherence. Compare with a routine baseline. | Park 2020 (21); Kim 2024 (22); Yamakado 2025 (23); Lee 2023 (24) |
| Systemic nutritional biomarker / serum 25-hydroxyvitamin D | Prognosis | Retrospective n=89; prospective n=91; preoperative → postoperative imaging; ≥1 year where reported | One positive association and one null cohort. | Exposure contrast, season, assay, and confounding differed. No supplementation evidence. | **Unclear** | Use an assay-calibrated multicenter cohort with season and confounder control. | Chen 2022 (25); Ryu 2015 (26) |
| Tissue biomarker / tendon degeneration and Bonar histology | Prognosis | Cohorts n=187, n=56, and 85 complete cases; intraoperative biopsy → 6–12-month imaging | One cohort was positive. Two cohorts were null. | Invasive sampling. Semiquantitative scoring. Few failures in null studies. | **Unclear** | Fix the sampling site. Blind scoring. Test inter-reader reliability. Replicate the same assay. | Tokunaga 2023 (37); Wang 2023 (38); Sethi 2018 (39) |
| Tissue biomarker / muscle fat, atrophy, and fibrosis histology | Prognosis | Prospective biopsy cohort n=53; 7 MRI retears; minimum 1 year; revision-free mean 38.8 months | No significant association with MRI retear or revision, assessed separately. | Only seven retears occurred, limiting precision. The assay was invasive, and retear and revision were evaluated as distinct outcomes. | **Unclear** | Evaluate a standardized assay in a larger prospective cohort and define the intraoperative decision it is intended to inform. | Ruderman 2026 (40) |
| Genetic biomarker / TNC haplotype and ESRRB rare variant | Prognosis | Candidate-gene cohorts n=302 and n=72; perioperative DNA → ≥1-year US/MRI | Candidate associations were reported in separate cohorts. | No same-variant replication. Ancestry restriction. Rare variants. | **Partial** | Preregister ancestry-aware replication and adjust for routine predictors. | Kluger 2017 (35); Tashjian 2016 (36) |
| Transcriptomic biomarker / RNA-seq and NanoString pathway signatures; mechanistic discovery | Prognosis candidate | n=36; n=24 repairs + 9 controls; n=25; intraoperative tendon → postoperative imaging/composite; follow-up timing varied across studies and was not uniformly reported. | Pathway and cluster differences were observed. No locked classifier emerged. | Small samples. Morphology confounding. Batch effects. Invasive specimen. | **Partial** | Prespecify a parsimonious signature. Perform batch-robust independent validation. | Eager 2021 (41); Tashjian 2020 (42); Lynskey 2026 (43) |
| Gene-expression biomarker / MMP-3 and TIMP-1; mechanistic discovery | Prognosis candidate | Prospective tissue study n=24; 6 retears; intraoperative tendon → ≥1-year MRI | Expression was higher among retears. | Very few events. Multiple testing. No threshold or adjusted model. | **Partial** | Test a prespecified targeted assay in a powered cohort with a routine benchmark. | Gotoh 2013 (44) |

Notes to Supplementary Table S1.

Evidence-profile status definitions: Established = consistent direct evidence for the stated intended use, generally from at least two independent cohorts; Partial = direct evidence supports at least one necessary component, but replication or another essential requirement is missing; Unclear = materially conflicting findings or insufficient reporting; Not demonstrated = no included study directly supports any necessary component of the stated use.

1. Supplementary Table S1 presents representative human evidence selected to cover the main evidence classes and intended uses, including positive, negative, and conflicting findings. Each Status applies only to the stated intended use. A profile may summarize one study or a small group of studies evaluating the same candidate and use.

2. When a profile informs more than one intended use, the Evidence-profile status cell reports each use separately. This column summarizes the maturity of direct evidence for the stated use; it is not a five-dimension readiness profile. The dimension-specific ratings in Table 1 were derived from all information in the relevant rows rather than from this column alone.

3. “Independent validation” requires a prespecified and unchanged candidate, measurement process, model, or threshold evaluated in a new population or setting. Split-sample evaluation or within-cohort modification is not independent validation.

4. “Added value” requires comparison with a prespecified benchmark containing routine clinical and conventional imaging predictors in the same evaluation cohort.

5. “Treatment-effect prediction” requires a prespecified candidate-by-treatment interaction. No qualifying evidence was identified among the included studies.

6. Biomarker and context-of-use terminology follows BEST (6). Model and radiomics evaluation is informed by TRIPOD+AI, PROBAST+AI, and current radiomics guidance (7–9).

7. BMD, bone mineral density; CEUS, contrast-enhanced ultrasound; MRI, magnetic resonance imaging; ROI, region of interest; US, ultrasonography; UTE, ultrashort echo time.

8. Reference numbers correspond to the main manuscript.

* Evidence-base descriptions and numerical estimates are study-specific and should not be compared directly across heterogeneous designs, endpoints, and follow-up windows.
